# The Basic Characteristics of the Pentraxin Family and Their Functions in Tumor Progression

**DOI:** 10.3389/fimmu.2020.01757

**Published:** 2020-08-18

**Authors:** Zeyu Wang, Xing Wang, Hecun Zou, Ziyu Dai, Songshan Feng, Mingyu Zhang, Gelei Xiao, Zhixiong Liu, Quan Cheng

**Affiliations:** ^1^Department of Neurosurgery, Xiangya Hospital, Central South University, Changsha, China; ^2^National Clinical Research Center for Geriatric Disorders, Changsha, China; ^3^Department of Neurosurgery, West China Hospital, Sichuan University, Chengdu, China; ^4^Department of Clinical Pharmacology, Xiangya Hospital, Central South University, Changsha, China

**Keywords:** C-reactive protein, serum amyloid P component, the long pentraxins, pentraxin 3, tumor

## Abstract

The pentraxin is a superfamily of proteins with the same domain known as the pentraxin domain at C-terminal. This family has two subgroups, namely; short pentraxins (C-reactive protein and serum amyloid P component) and long pentraxins (neuronal pentraxin 1, neuronal pentraxin 2, neuronal pentraxin receptor, pentraxin 3 and pentraxin 4). Each group shares a similar structure with the pentameric complexes arranged in a discoid shape. Previous studies revealed the functions of different pentraxin family members. Most of them are associated with human innate immunity. Inflammation has commonly been associated with tumor progression, implying that the pentraxin family might also participate in tumor progression. Therefore, we reviewed the basic characteristics and functions of the pentraxin family and their role in tumor progression.

## Introduction

The pentraxin family is a superfamily of protein that share the same domain and are made from monomers arranged in pentameric structures with a discoid shape (1). The members of the family are characterized by a 205 amino acids (AA) long conserved sequence located at C-terminal called the pentraxin domain. Members of the pentraxin family share a similar 8 AA (His-x-Cys-x-Ser/Thr-Trp-x-Ser, in which x represent any AA) long conserved sequence called the pentraxin signature within the pentraxin domain (2). Based on the length of the protein sequence, the pentraxin family can be classified into two subfamilies: the short and long pentraxins. The short pentraxins are comprised of C-reactive protein (CRP) and serum amyloid P component (SAP), whereas the long pentraxins are composed of neuronal pentraxin 1(NPTX1), neuronal pentraxin 2 (NPTX2), neuronal pentraxin receptor (NPTXR), pentraxin 3 (PTX3), and pentraxin 4 (PTX4) (3). The long pentraxins are approximately twice the size of the short pentraxins with an un-related long N-terminal sequence. This structure variability of family members could explain their function difference.

In the past decades, studies have revealed the functions of specific members of the pentraxin family. For example, the member of the short pentraxin family, CRP, and SAP were previously reported as mediators in human immune system regulation (4–6). Their functions in immune regulation include acting against pathogen invasion, removing mutant cells, and triggering inflammation. The neuronal pentraxins are involved in the development of the central nervous system and neurodegenerative diseases (7). PTX3 not only participates in immune system activation but also affects tumor progression (8, 9).

Chronic inflammations such as chronic atrophic gastritis and cervical intraepithelial neoplasia have been recognized as precancerous lesions (10). Therefore, inflammation is closely associated with tumor progression, including tumorigenesis, metastasis. Reactive oxygen/nitrogen species (ROS/RNS) produced by immune cells and epithelial cells fight against microbial invasion and eliminate the mutant cell. However, they can cause cell dysfunction and promote tumorigenesis (11, 12). Tumors induce inflammation by either producing antibodies or rejecting immunocytes infiltration to avoid immune system surveillance (13). Additionally, the tumor microenvironment facilitates the growth of cancer cells, metastasis, and enhances drug resistance (14). It is necessary to explore the mechanisms through which inflammation and tumor microenvironment enhances tumor progression.

Several reports have also been documented on the role of the pentraxin family in tumor progression. Likewise, several receptors and pathways have been proposed that could be associated with the mechanisms employed by the pentraxin family in mediating tumor progression. Most members of the pentraxin family can activate the PI3K/AKT/mTOR pathways, thereby interfering with the normal cell- cycle. The neuronal pentraxins and PTX3 possess specific unique receptors that mediates tumor progression (15). In this review, we have summarized the basic characteristics and functions of each pentraxin family member and highlighted the existing connection between their structures and specific roles in tumor progression.

## C-Reactive Protein

The human C-reactive protein (CRP) gene is located on the chromosome 1q23.2 (16), and has a length of 1.8-kb. It consists of 0.1 kb at untranslated region (UTR) in the 5′ terminal and a 1.2 kb pair UTR region in the 3′ terminal, with two exons separated by an intron. The first exon encodes 18 AA signal peptide and the first two amino acids, whereas, the second exon encodes the rest AA (16). The X-ray derived structures of CPR are pentameric with five subunits arranged in a discoid shape. Each unit contains 206 AA with two anti-parallel b-sheets appearing as a flattened b-barrel with a jellyroll topology. The two sides of its discoid have distinct functions. The Ca^2+^ binds to the “A” side and activates the classical complement pathway and phagocytosis by interacting with C1q and Fcγ receptor, respectively (17, 18). The “B” side, CRP recognizes phosphocholine (PCh), a bacterial cell wall component, and eliminates the pathogen (19). The CRP also binds to soluble control protein factor H regulating the alternative-pathway amplification and C3 convertase (18). The secretion of CRP by hepatocytes can be stimulated by the IL6 and IL1 (20), which enhances innate immunity by triggering inflammation and neutralizing pathogen (18, 21).

### CRP and Tumor

CRP activates various signaling pathways by binding to the Fcγ receptor (22, 23), which links it with inflammation (17). The PI3K/AKT/mTOR signaling pathway is associated with tumor cell proliferation, metabolic reprogramming, apoptosis, and metastasis (24). For instance, the CRP arrest cell-cycle at the sub G1 phase by negatively regulating the PI3K/AKT/mTOR signaling pathway in myeloid leukemia (25) and tongue squamous cell carcinoma (26) thus promoting tumor progression. It has been reported that interaction between CRP and Fcγ receptor I facilitate tumor cell metastasis in breast cancer (23). Furthermore, CRP targets to the p38/MAPK pathway causing lytic bone lesions (27) and activates the PI3K/AKT/mTOR and the ERK/NF-κB pathway thereby inhibiting tumor cell apoptosis via the Fcγ receptor II in multiple myeloma (22).

Previous reports showed that CRP is a clinical marker for infection and has a regulatory role in innate immunity (17, 28). The IL-6/JAK/STAT signaling pathway has been reported to enhance CRP expression in glioblastoma (29), clear cell renal cell carcinoma (30) and gastroesophageal cancers (31). Moreover, the pathway enhances the formation of CRP-mediated tumor microenvironment by activating tumor-associated macrophages (30) and tumor angiogenesis (32, 33). CRP regulates cell apoptosis and cell-cycle in clear cell renal cell cancer (34). Notably, inhibiting CRP expression by targeting IL-1 can prolong overall survival time for patients with multiple myeloma (35).

**Figure 1:**
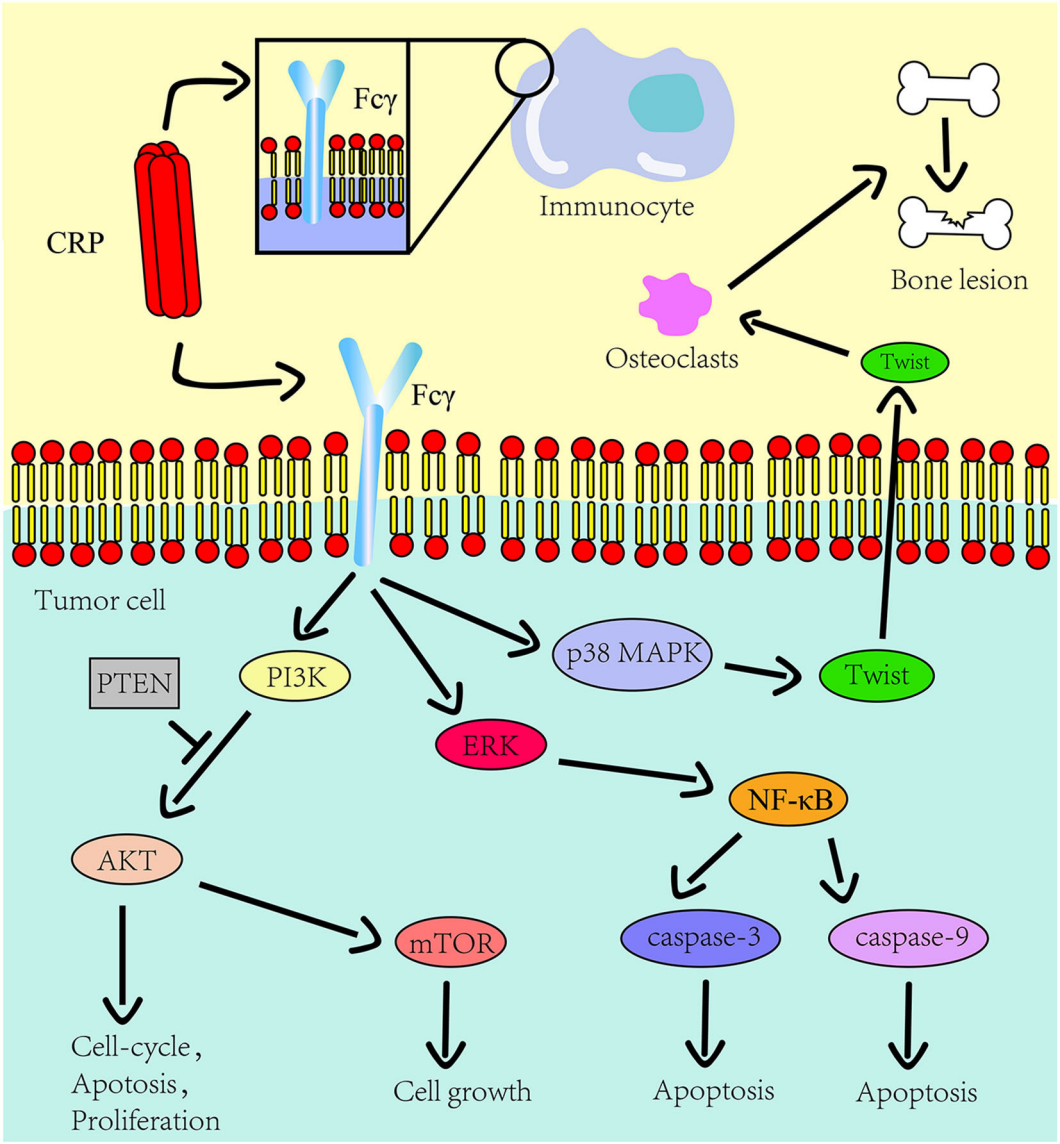
The role CRP on the innate immune system and tumor progression. The Fcγ receptor is expressed on the cell surface of immunocyte and multiple myeloma cells. CRP binds to Fcγ receptor to promote inflammation and tumor progression. The downstream pathways of this receptor in tumors include the PI3K/AKT/mTOR pathway, the ERK/NF-κB pathway and the p38/MAPK pathway. CRP regulates the expression of osteolytic cytokines in myeloma cells through p38 MAPK-Twist signaling.

Multiple studies have, therefore, confirmed that CRP could be used as a prognosis factor (36–38). This has been reported in different tumors including breast cancer (39), prostate cancer (40), non-small cell lung cancer (41), hepatocellular carcinoma (42), cervical cancer (43), head and neck squamous cell carcinoma (44), diffuse large B-cell lymphoma (45) and osteosarcoma (46). The specific mechanisms involved are still lacking. Controversies have also emerged in determining the role of CRP in pancreatic cancer (47, 48) and colorectal cancer (49–51). Considering that CRP also affects tumor progression, more effort in explicating its role in the tumor might be beneficial in understanding the connection between tumor and human innate immunity (Figure 1).

## Serum Amyloid P Component

**Figure 2:**
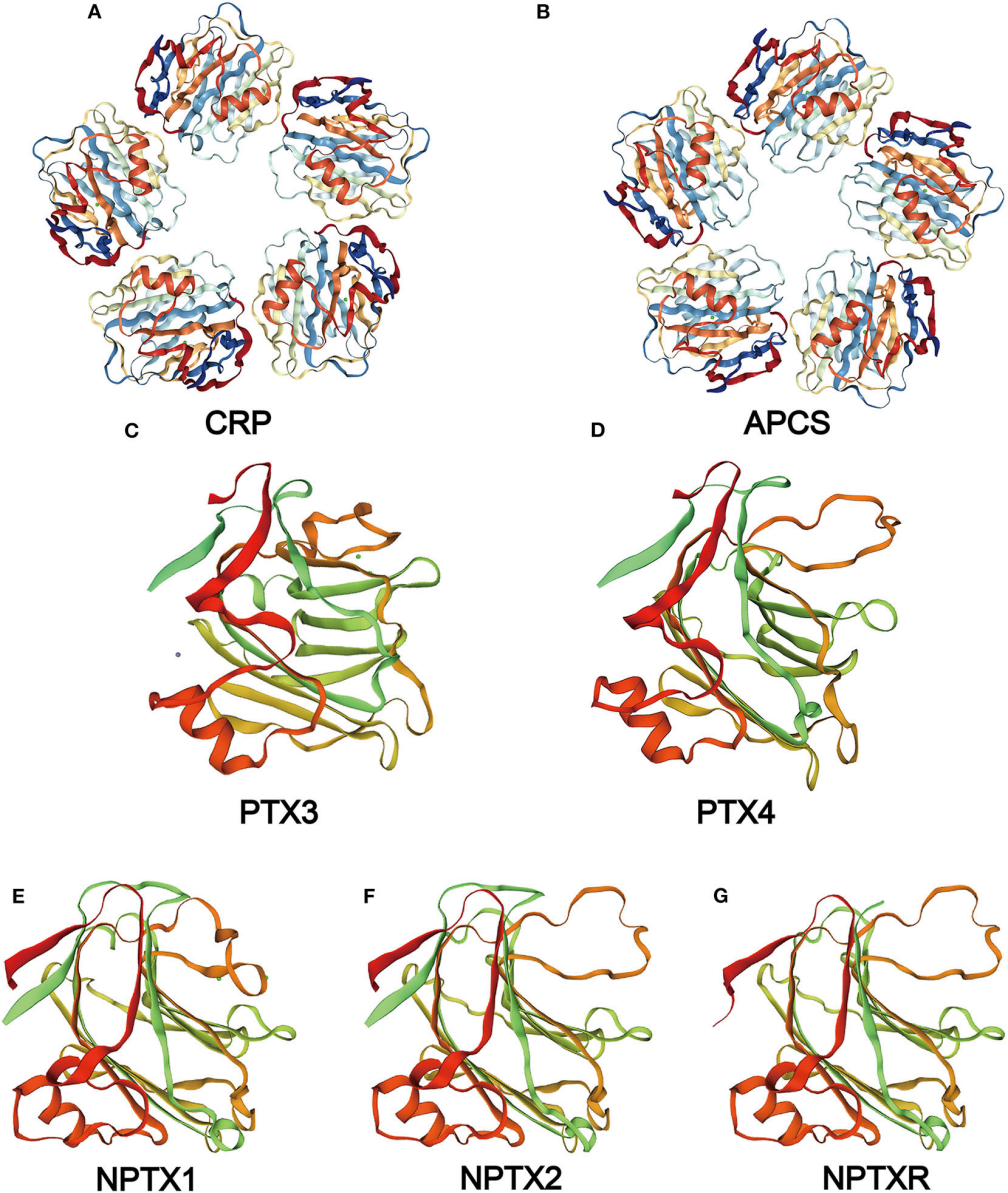
Structure of the pentraxin family members. Protein structure of CRP **(A)** and SAP **(B)** from the PDB website and the hypothetical structure of the full monomer (C-terminal domain and N-terminal domain) of PTX3 **(C)**, PTX4 **(D)**, NPTX1 **(E)**, NPTX2 **(F)**, NPTXR **(G)** from the Swiss database.

**Figure 3:**
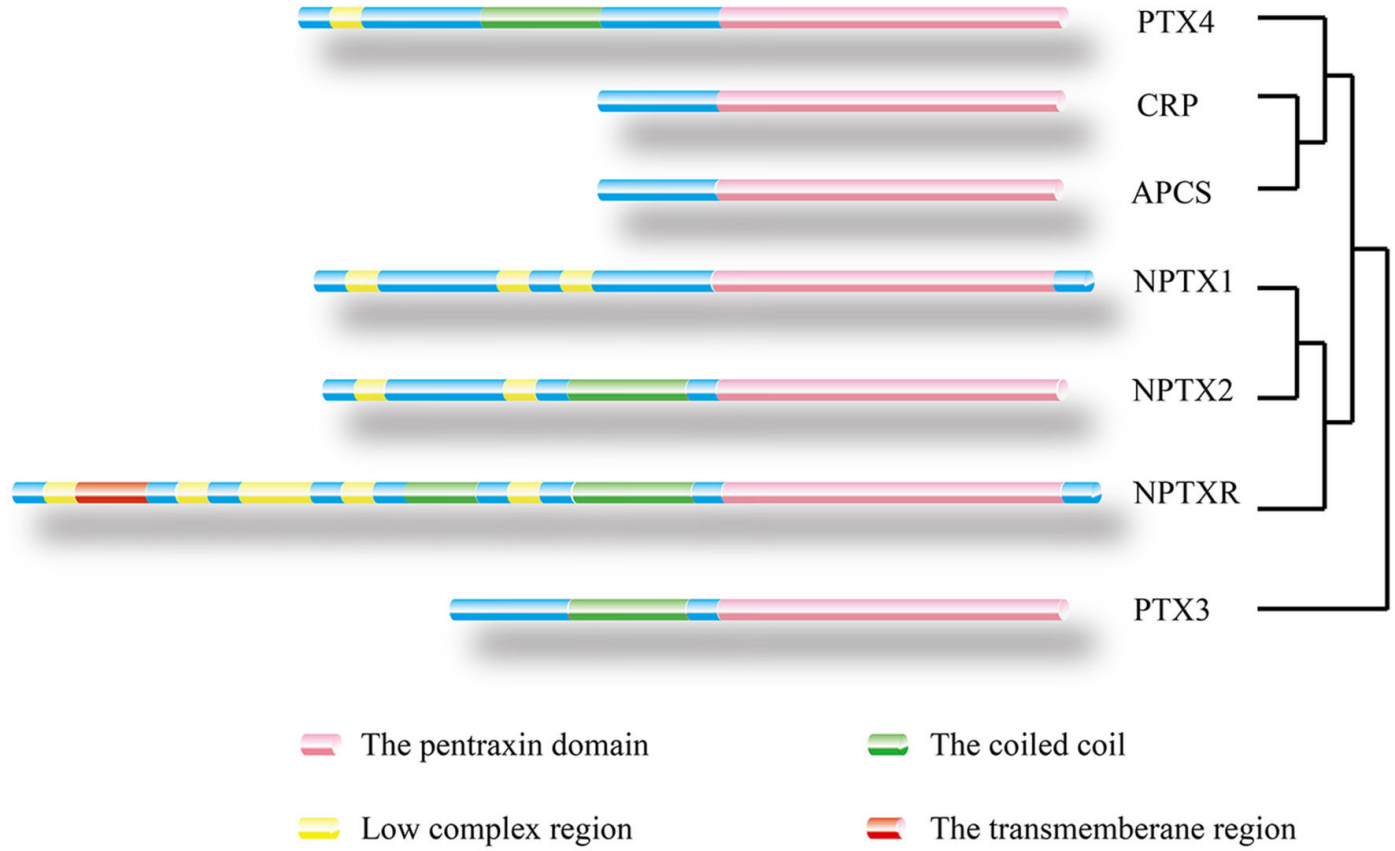
Location of protein domain for each family member and their sequence homology. The outcome was predicted by the Pfam database. Pink box: Pentraxin domain. Green box: Coiled coil. Yellow box: Low complex region. Red box: Transmembrane region. A homology tree based on the similarity of the protein sequence of members of the pentraxin family is generated.

The serum amyloid P component (SAP) gene, identified as a close CRP paralog, is also localized on the chromosome 1q23.2 and shares the same gene architecture (52). The gene is approximately 1.1 kb long with 0.1 kb 5′ UTR and 0.15 kb 3′ UTR. The SAP structure is similar to CRP except that its subunits consist of 204 AA and has a slight difference at the calcium-binding site (4, 53, 54). In the absence of calcium, SAP form decamers composed of two pentamers facing each other (4). The two ligands of SAP, deoxyadenosine 5′-monophosphate (dAMP), and the 4,6-pyruvate acetal of β-D-galactose (MoβDG), bind calcium and amyloid fibrils, respectively (55). To distinguish structure and gene sequence of short pentraxins, we archived data on the 3D structure for CRP and SAP from the PDB web portal (https://www.rcsb.org/, Figures 2A,B). The prediction of their domain was inferred from the Pfam database (http://pfam.xfam.org/, Figure 3) and the Genecard database (https://www.genecards.org/, Table 1).

**Table 1:** The basic characteristics of two short pentraxins, CRP and SAP.

**Characteristics**	**CRP**	**SAP**
Genomic location	1q23.2	1q23.2
Gene sequence	1.8 kb in length, 0.1 kb of 5' UTR, 1.2 kb of 3' UTR	1.1 kb in length, 0.1 kb of 5' UTR, 0.15 kb of 3' UTR
Domain structures	Pentraxin-related, ConA-like_dom_sf, Pentaxin_CS	Pentraxin-related, ConA-like_dom_sf, Pentaxin_CS
Top tissue expression	Liver	Liver
Signaling pathway	PI3K/Akt signaling pathway (25), JAK/STAT signaling pathway (35)	PI3K/Akt/ERK signaling pathway (56)

*CRP, C-reactive protein; SAP, serum amyloid P component; UTR, un-translated region; The domain structures were obtained from the Genecard database*.

Like CRP, SAP is also secreted by hepatocytes and mediates innate immunity by interacting with the complement system and the Fcγ receptor (17). This decreases neutrophil adhesion, inhibits neutrophil spreading, regulates macrophage activation (56), and inhibits fibrocyte differentiation (57). Besides, SAP is involved in immunological tolerance by binding to DNA or chromatin resulting from necrosis and apoptosis cell (58). Furthermore, SAP binds to amyloid fibrils through MoβDG, thereby causing amyloidosis disease (4–6). SAP is also associated with tuberculosis (59) and sickle cell disease (60), but the specific mechanisms are unknown. Despite limited reports on the role of SAP in tumors, SAP is considered a prognosis factor in non-small cell lung cancer (61). The highly structural homology between SAP and CRP suggests that SAP has the potential to mediate tumor progression through the Fcγ receptor.

## The Neuronal Pentraxins

Neuronal pentraxin 1 (NPTX1 or NP1) is a 47–50 kDa secreted glycoprotein mainly expressed in neurons. The NPTX1 gene is located on chromosome 17q25.3. Its cDNA clones sequence is made up of a 150 bp 5′UTR, a 1.3 kb coding region, and a 3.6 kb 3′UTR with four introns (62). NPTX1 contains three main domains: a putative ligand- and calcium-binding site, the pentraxin domain, and an Asn-linked glycosylation site (62). Three NPTX1 domains, including the Pentraxin-related domain, the Pentraxin_CS (Pentraxin, conserved site), and glucanase domain superfamily (ConA-like_dom_sf) are speculated from the Genecard database.

Neuronal pentraxin 2 (NPTX2 or NP2, also known as apexin/p50 in guinea pig or narp in rat), is a ~47 kDa secretory glycoprotein with 431 AA. It is expressed in various tissues of the brain, testicle, pancreas, and skeletal muscle (1). The human NPTX2 gene is located on chromosome 7q22.1. Its cDNA sequence is made up of a 1.3 kb coding region and 1.2 kb 3′-UTR with four introns (1). Domains of NPTX2 are the same as those of NPTX1, according to the speculation from the Genecard website.

Neuronal pentraxin receptor (NPTXR or NPR) is an ~53 kDa type-II transmembrane protein with 500 AA and is mostly expressed in the brain. It is the only pentraxin family member anchored to the cell membrane by a putative N-terminal transmembrane domain. The receptor binds tightly to its ligands, such as taipoxin, TCBP49, NPTX1, and NPTX2, and activates different downstream signal transduction processes (63). In the human genome, the NPTXR gene is located on chromosome 22q13.1 and has the longest cDNA clones sequence containing a 3.9 kb 3′ UTR and a 1.5 kb open reading frame (63). From the Gencard database, this protein consists of two main domains, Pentraxin-related domain and glucanase domain superfamily (ConA-like_dom_sf). The N-terminal structure of the neuronal pentraxins is unrelated to other known human protein structures (64). Therefore, multiple online databases were used to generated detailed information about the structures of the neuronal pentraxins. The 3D structures of monomer were referenced from the Swiss database (https://www.swissmodel.expasy.org/, Figures 2E–G), and their domains were projected from the Pfam (Figure 3) and the Genecard databases (Table 2).

**Table 2:** The basic characteristics of the long pentraxins.

**Characteristics**	**NPTX1**	**NPTX2**	**NPTXR**	**PTX3**	**PTX4**
Genomic location	17q25.3	7q22.1	22q13.1	3q25.32	16p13.3
Sequence features	150 bp of 5' UTR, 1.3 kb coding sequence, 3.6 kb 3' UTR	1.3 kb coding sequence, 1.2 kb 3'-UTR	5.5 kb in length, 3.9 kb of 3' UTR, 1.5 kb open reading frame	68 bp of 5' UTR, 650 bp of 3' UTR,	–
Domain structures	Pentraxin-related, Pentraxin_CS, ConA-like_dom_sf	Pentraxin-related, Pentraxin_CS,ConA-like_dom_sf	Pentraxin-related, ConA-like_dom_sf, N-terminal transmembrane domain	Pentraxin-related, Pentraxin_CS, ConA-like_dom_sf	Pentraxin-related, Pentraxin_CS,ConA-like_dom_sf
Top tissue expression	Brain	Brain, liver, testis, skeletal muscle, heart, pancreas	Brain	Monocytes, macrophage, fibroblasts, epithelial cells	Thymus, spleen, small intestine, liver
Signaling pathway	HIF-1 signaling pathway (65), IRS-1/PI3K/Akt signaling pathway (66), JNK and GSK3 signaling pathways (67), Rb/E2F pathway (68), Nodal and BMP signaling pathway (69)	Wnt/β-catenin signaling pathway (70), p53/PTEN/Akt/NF-κB signaling pathway (71)	–	Akt/NF-kB signaling pathway (9), JNK signaling pathway (72), IL-6/Stat3 signaling pathway (73), PI3K signaling pathway (74)	–

*NPTX1, neuronal pentraxin 1; NPTX2, neuronal pentraxin 2; NPTXR, neuronal pentraxin receptor; PTX3, pentraxin 3; PTX4, pentraxin 4; UTR, un-translated region; Domain structure were obtained from the Genecard database*.

### The Function of the Neuronal Pentraxins

The neuronal pentraxins have different functions in the development of the central nervous system (7) such as mediation of neural differentiation (69), synaptogenesis (75) and synapse plasticity (76, 77). Abnormal expression of the neuronal pentraxins has been reported in some mental diseases such as bipolar disorder (78), central precocious puberty (79), anxiety (80), depression (80), childhood-onset mood disorders (81) and schizophrenia (82). The neuronal pentraxins are associated with neurodegenerative diseases, including Alzheimer's disease (AD) (83, 84) and Parkinson's disease (PD) (85, 86). Researchers concluded that NPTXR protein in cerebrospinal fluid is a novel potential biomarker of AD progression and could have important utility in assessing treatment success in clinical trials (83), and NPTX1 could significantly contribute to the pathogenesis of PD (87). Moreover, only NPTX1 among all the neuronal pentraxins participates in inflammation by inducing mitochondria dysfunction (65, 67). Despite the neuronal pentraxins contains the pentraxin domain like the short pentraxins, few studies have classified their roles in human innate immunity which might result from the difference in their tertiary structure.

### The Neuronal Pentraxins in Cancer

Several pathways were identified as potential mechanisms through which the neuronal pentraxins promote tumor progression. NPTX1 and NPTX2 were reported to cause dysfunction of the PI3K/AKT/mTOR pathway thereby affecting tumor progression in glioma (66), gastrointestinal stromal tumors (GIST) (88) and subependymal giant cell astrocytoma (89). Additionally, NPTX2 promotes tumor cell proliferation and metastasis by activating the NF-κB pathway (71, 90) and the Wnt/β-catenin pathway (70). It also induces tissue edema via an independent pathway from the classical VEGF-relate pathway (91). The dysfunction of the PI3K/AKT/mTOR pathway interferes with the normal cell-cycle and causes tumorigenesis. Similarly, NPTX1 and NPTX2 can inhibit cyclin A2 and CDK2 through the Rb/E2F signaling pathway (68), respectively, thus inducing G0/G1 arrest in pancreatic cancer (92, 93). Overexpression of NPTX2 has been identified as a prognosis factor in clear cell renal cell carcinoma, and its interaction with AMPA-selective glutamate receptor-4 affects tumor cell viability and metastasis (94, 95).

Abnormal expression of the neuronal pentraxins has been reported in different tumors such as cervical carcinoma (96), primary lung cancer (97), Ewing sarcoma (98), neuroblastoma (99), small cell lung cancer (100) and neuroblastoma (99). Another study has reported an increase in NPTX1 expression in pancreatic cancer after treatment with metformin and aspirin (101). On the contrary, a low level of NPTX2 showed better response to neoadjuvant chemoradiation (CRT) treatment in rectal adenocarcinomas (102).

The neuronal pentraxins are crucial in the central nervous system development when they interact with the AMPA receptor. The AMPA receptor has been proved to be associated with tumors (103, 104). Therefore, the AMPA receptor is a potential linkage between the neuronal pentraxins and tumor development as opposed to other members of the pentraxin family. Conclusively, the above-reviewed studies did not reveal the explicit mechanism through which the neuronal pentraxins affect tumor progression; however, they form the basis for in-depth studies on the existing association between the two.

## Pentraxin 3 and Pentraxin 4

Pentraxin 3 (PTX3) and pentraxin 4 (PTX4) are characterized as long pentraxins. The PTX3 gene is located on chromosome 3q25 and has three exons and two introns (105). The three exons encode the leader peptide, an N-terminal domain, and the pentraxin domain, respectively (106). The N-terminal domain of PTX3 and PTX4 is not related to any known protein. However, based on previous research, PTX3 has a putative N-terminal domain that shows structural similarity to the mannose-binding protein and the surfactant proteins (107). PTX3 is secreted by different cell types that include dendritic cells, macrophages, and fibroblasts (108).

PTX4 gene is located on chromosome 16p13.3 and consists of three exons. However, the human PTX4 cDNA sequence and its first exon failed to amplify because it was different from the sequences in the various database (109). The sequence analysis of the protein showed a highly structural homology between PTX4 and the short pentraxins (Figure 2D). This indicates that PTX4 might be playing specific roles in innate immunity or tumor progression. To compare the PTX3 and PTX4 structures, their 3D structures were predicted from the Swiss database (https://www.swissmodel.expasy.org/, Figures 2C,D), and their domain predicted from the Pfam (Figure 3) and the Genecard databases (Table 2).

### Functions of PTX3 and PTX4

From the previous study, an increase in blood PTX3 concentration serves as a monitor of inflammation initiation. The maximal PTX3 level increased slightly earlier than the CRP level (110), suggesting that PTX3 could be a highly sensitive inflammation-related factor. The interaction between PTX3 and the complement system has broad implications in host defense against microbial infections, regulation of the inflammatory reaction, and removal of dead cells. PTX3 is actively involved in the complement pathways activation (111). For example, the classical activation cascade may be initiated when PTX3 binds to C1q via the Fcγ receptor III once the latter is bound on a microbial surface (64, 112, 113). However, the process can be inhibited if the interaction occurs in the fluid-phase (114).

Furthermore, the N-terminal domain of PTX3 enhances tissue repair and remodeling functions (115). A study reported that PTX3 inhibited interstitial fibrosis in acute renal injury (73), indicating its role in an extracellular matrix formation. The N-terminal domain also combines with FGF2 to mediate angiogenic activity (116). For PTX4, there is currently no evidence supporting its functions in innate immunity or tumor progression.

### PTX3 and Cancer

PTX3 interacts with the PI3K/AKT/mTOR signaling pathway to induce tumor cell proliferation, apoptosis and metastasis in lung cancer (9), head and neck squamous cell carcinoma (74) and breast cancer (117). PTX3 also inhibits cell proliferation and tumor metastasis by modulating the expression of protein related to the G2/M phase cell-cycle in cervical cancer (118). Furthermore, it arrests cell-cycle at the G0/G1 phase by stimulating the secretion of p21 protein in glioma (119).

Notably, we reported that PTX3 interacts with the fibroblast growth factor-2 (FGF2)/FGF receptor (FGFR) system that mediates the epithelial-mesenchymal transition (EMT) through its N-terminal domain (120). Through this system, PTX3 inhibits tumor metastasis, tumor growth and tumor angiogenesis in melanoma (8), breast cancer (121), prostate cancer (122) and multiple myeloma (123). Besides, PTX3 binds to fibroblast growth factor-8b receptor (FGF8b) and inhibits tumor cell proliferation in steroid hormone-regulated tumors (124). Compared to other pentraxin family members, the PTX3 is highly associated with the FGFR system.

Abnormal PTX3 expression was also observed in different tumors, including glioma (125), esophageal squamous cell carcinoma (126), pancreatic cancer (127), gastric cancer (128), colorectal cancer (129), leiomyosarcoma and desmoid tumors (130). Previous studies have shown that the activation of Fcγ receptor promotes tumor progression (131, 132). Of note, Fcγ receptor expression on NK cells modulates tumor response to immunotherapy (133), and PTX3 can exert its function in human immunity by interacting with this receptor. However, no current research has bridged PTX3 and tumor progression through the Fcγ receptor. Therefore, the multifaceted role of PTX3 in cancer requires further comprehensive study.

## Overall Survival Analysis Prediction

We conducted the overall survival analysis of the pentraxin family to establish the relationship between the tumor and the pentraxin family members using the Gepia web portal (http://gepia.cancer-pku.cn/, Supplementary Table).

**Figure 4:**
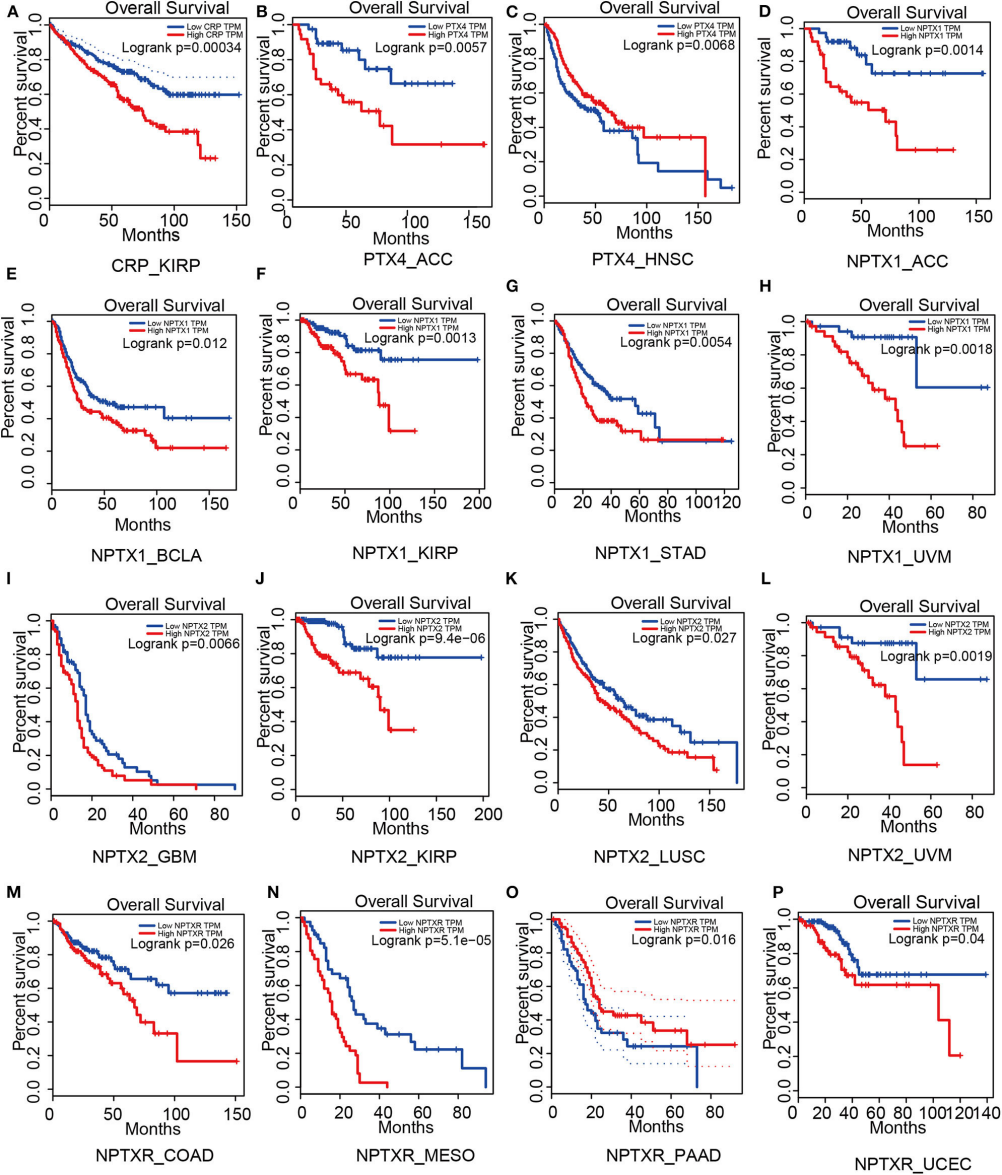
Survival analysis based on members of the pentraxin family from the Gepia website. Outcome of survival analysis predicted by the Gepia database including CRP **(A)**, PTX4 **(B,C)** and the neuronal pentraxins **(D–P)**.

For short pentraxins, low expression of CRP showed better survival outcomes in kidney renal papillary cell carcinoma (KIRP) than high CRP expression (Figure 4A). There was no significant difference in survival outcome between the high and low expression of SAP. The expression levels of PTX4, with similar protein sequence as short pentraxins, showed a significant difference in survival outcome in adrenocortical carcinoma (ACC) and head and neck squamous cell carcinoma (HNSC) (Figures 4B,C).

We revealed that low NPTX1 expression improved the survival outcomes in patients with adrenocortical carcinoma (ACC), urothelial bladder carcinoma (BCLA), kidney renal papillary cell carcinoma (KIRP), stomach adenocarcinoma (STAD) and uveal melanoma (UVM), (Figures 4D–H). Patients that showed high expression of NPTX2 frequently exhibited worse survival outcomes for glioblastoma multiforme (GBM), kidney renal papillary cell carcinoma (KIRP), lung squamous cell carcinoma (LUSC) and uveal melanoma (UVM), (Figures 4I–L). On the other hand, overexpression of NPTXR predicted worse survival outcomes for colon adenocarcinoma (COAD), mesothelioma (MESO), and pancreatic adenocarcinoma (PAAD) and better survival outcome in uterine corpus endometrial carcinoma (UCEC) (Figures 4M–P).

**Figure 5:**
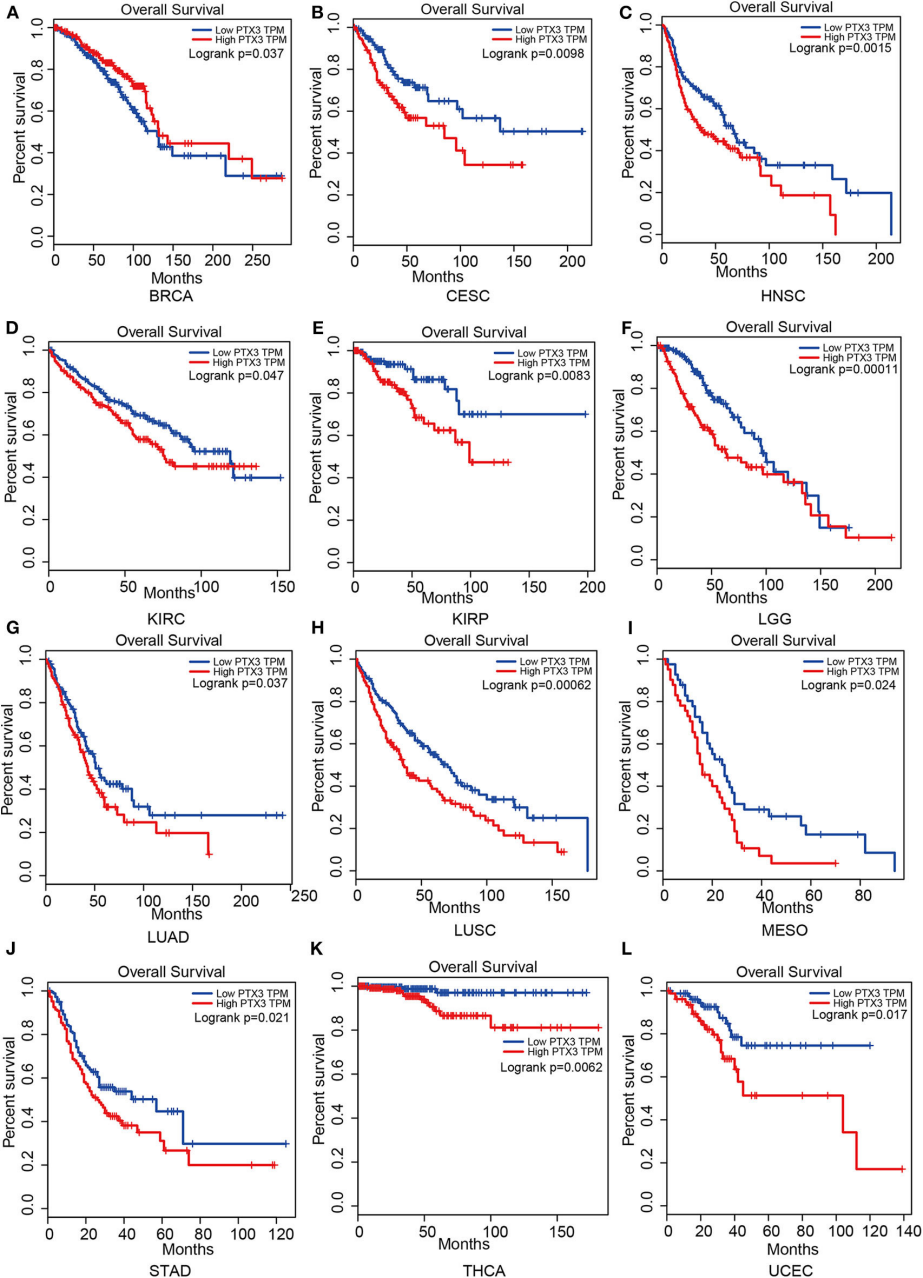
Impact of PTX3 on overall survival and tumor phenotypes based on the Gepia dataset. **(A)** Breast invasive carcinoma. **(B)** Cervical squamous cell carcinoma and endocervical adenocarcinoma. **(C)** Head and Neck squamous cell carcinoma. **(D)** Kidney renal clear cell carcinoma. **(E)** Kidney renal papillary cell carcinoma. **(F)** Brain Lower Grade Glioma. **(G)** Lung adenocarcinoma. **(H)** Lung squamous cell carcinoma. **(I)** Mesothelioma. **(J)** Stomach adenocarcinoma. **(K)** Thyroid carcinoma. **(L)** Uterine Corpus Endometrial Carcinoma. The overall survival of tumor that cannot be predicted by PTX3 expression is not listed.

Therefore, PTX3 is considered a promoter in tumor progression since its overexpression resulted in worse survival outcomes in invasive breast carcinoma (BRCA), cervical squamous cell carcinoma and endocervical adenocarcinoma (CESC), head and neck squamous cell carcinoma (HNSC), kidney renal clear cell carcinoma (KIRC), kidney renal papillary cell carcinoma (KIRP), brain lower-grade glioma (LGG), lung adenocarcinoma (LUAD), lung squamous cell carcinoma (LUSC), mesothelioma (MESO), stomach adenocarcinoma (STAD), thyroid carcinoma (THCA) and uterine corpus endometrial carcinoma (UCEC), (Figure 5).

The protein structure variability sheds light on the prognosis prediction ability of members of the pentraxin family. The expression of PTX3 affects multiple tumor types compared to other members of the pentraxin family, implying a potential association between its putative N-terminal domain and tumor progression. PTX4 shares a similar protein structure with the short pentraxins, and they are both poor predictors of survival outcomes of patients suggesting that they mainly concentrate on monitoring inflammation. However, this deduced relationship between protein structure and their prognosis capability requires further confirmation.

## Conclusions

**Figure 6:**
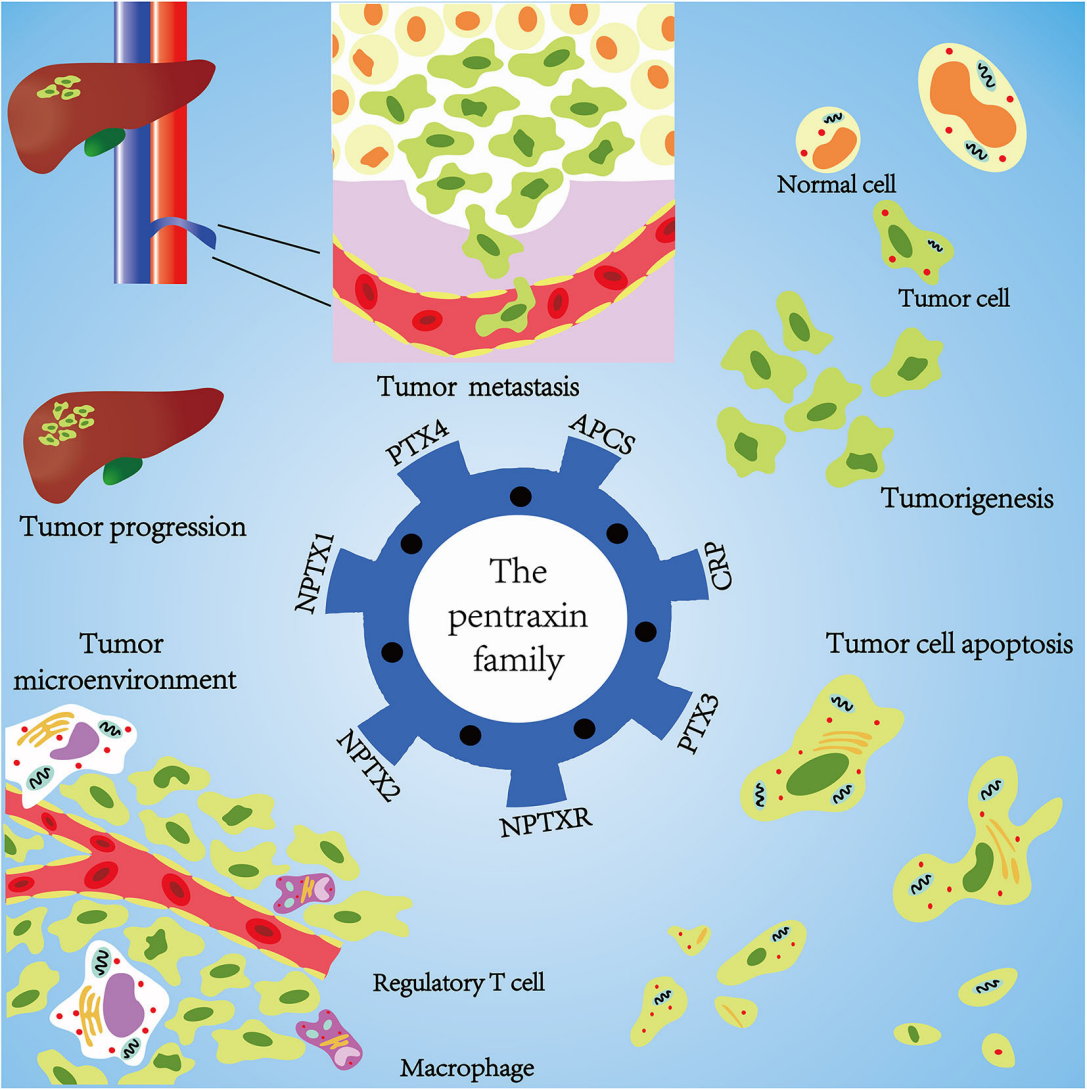
Mechanisms of pentraxin family in tumor progression. At the core is the pentraxin family and its seven members. The pentraxin family mainly participates in tumor metastasis, tumorigenesis, tumor cell apoptosis, tumor cell proliferation, and regulation of tumor microenvironment.

Emerging evidence confirms that the pentraxin family is associated with tumor progression by affecting tumor proliferation, mediating tumor cell apoptosis, inducing tumor metastasis, and promoting tumor tissue edema (Table 3, Figure 6). The tumor microenvironment is an extremely complex network consisting of cancer-associated fibroblasts, adipose cells, immunocytes, new-born vessels, and extracellular matrix. The pentraxin family promotes the formation of tumor microenvironment by facilitating macrophage infiltration and stimulating cytokines secretion. The pentraxin family, therefore, plays an essential role in tumor progression, although the exact mechanisms are still unknown and require further in-depth studies.

**Table 3:** Role of pentraxins in the control of various tumors.

**Tumor**	**Gene**	**Expression**	**References**	**Mechanism**
Glioma	PTX3	Differ from low and high grade	(134)	Arrest cell cycle at the G0/G1 phase to affect glioma proliferation and metastasis.
Glioma	NPTX1	Increased	(66)	Promote tumor proliferation and metastasis via the IRS-1/PI3K/AKT signaling pathway
Glioma	NPTX2	Increased	(91)	Induce tumor tissue edema independent of the classical VEGF-relate pathway
Glioblastoma	CRP	Increased	(29)	Stimulate microglial cells to secret IL-1β which could induce tumor angiogenesis.
Glioblastoma	NPTX2	Decreased	(90)	Increase survival ratio through reducing NF-κB activity via inhibiting AKT by p53/PTEN-dependent pathway
Neuroblastoma	NPTX2	Increased	(92)	NPTX2 antagonist could reduce tumor progression
Neuroblastoma	NPTXR	Increased	(92)	Give NPTXR antagonist inhibit tumor progress
Subependymal giant cell astrocytoma	NPTX1	Decreased	(89)	After inhibiting the mTOR signaling pathway not only decrease tumor volume but also increase the expression of NPTX1
Colorectal cancer	NPTX2	Increased	(70)	Combine to frizzled class receptor 6(FZD6) which activate the Wnt/β-catenin signaling pathway to promote tumor growth and metastasis
Rectal adenocarcinomas	NPTX2	Decreased	(102)	Low level expression improve response to neoadjuvant chemoradiation (CRT) treatment
Colorectal cancer	NPTX1	Decreased	(68)	Inhibit cell proliferation by influence the combination of cyclin A2 and CDK2 and the Rb-E2F signaling pathway
Lung cancer	PTX3	Increased	(9)	Deglycosylased PTX3 observed suppress tumor migration via inactivating the PI3K/AKT and the NF-κB signaling pathway.
Non-small Cell Lung Cancer	SAP	Increased	(61)	SAP contributes to the clearance of apoptotic cells.
Breast cancer	CRP	Increased	(23)	Binds to Fcγ receptor I and to promote tumor metastasis.
Breast cancer	PTX3	Decreased	(124)	PTX3 inhibit tumor progression via combining to receptor of fibroblast growth factor-8b (FGF8b) and metastasis by activating the EMT process.
Clear cell renal cell cancer	NPTX2	Increased	(94)	Promote tumor viability and invasion via binding to AMPA-selective glutamate receptor-4
Clear cell renal cell cancer	CRP	Increased	(34)	Up–regulate the expression of ATG9B gene to inhibit tumor cell apoptosis and the formation of tumor microenvironment.
Head and neck cancer	PTX3	Increased	(135)	Affect tumor metastasis via the PI3K/AKT and the NF-κB signaling pathway.
Head and heck squamous cell carcinoma	CRP	Increased	(44)	Promote tumor cell proliferation, metastasis and angiogenesis through the PI3K/AKT signaling pathway.
Multiple myeloma	CRP	Increased	(25)	Binds to Fcγ receptor II to protect tumor and cause lytic bone lesions
Multiple myeloma	PTX3	Increased	(123)	Promote tumor cell proliferation, metastasis and angiogenesis through the PI3K/AKT signaling pathway.
Melanoma	CRP	Increased	(136)	Inhibit tumor angiogenesis, via FGF2/FGFR system, proliferation and apoptosis.
Melanoma	PTX3	Increased	(8)	Cause vitamin D deficiency.
Cervical cancer	PTX3	Increased	(118)	Inhibit tumor metastasis via FGF2/FGFR system which impair the EMT process
Gastroesophageal cancer	CRP	Increased	(31)	Modulate the G2/M phase cell-cycle related protein expression to affect cell proliferation.
Hepatocellular carcinoma	CRP	Increased	(137)	Promote tumor angiogenesis via influencing certain cytokines.
Myeloid leukemia	CRP	Increased	(25)	Its expression level is parallel with HBV activity while silence could promote tumor progression.
Pancreatic cancer	NPTX2	Decreased	(92)	Inhibits cell proliferation through the PI3K/AKT signaling pathway.
Prostate cancer	PTX3	Decreased	(122)	Inhibit tumor progression and migration decrease via inducing cell G0–G1 arrest and inhibiting cell apoptosis

*Data from merely abnormal expression or with lack of specific relationships are not mentioned*.

*CRP, C-reactive protein; SAP, serum amyloid P component; NPTX1, neuronal pentraxin 1; NPTX2, neuronal pentraxin 2; NPTXR, neuronal pentraxin receptor; PTX3, pentraxin 3; PTX4, pentraxin 4*.

Protein structure of short pentraxins and their role in the immune system has highly been explored compared to the long pentraxins. It has been reported that PTX3 and NPTXR, for example, have an additional N-terminal domain other than the known pentraxin domain and exhibit different functions. Each pentraxin family member has their specific unique functions, for instance, SAP binds to amyloid fibrils and the neuronal pentraxins and extensively participates in the central nervous system development. Furthermore, the pentraxin family affects tumor progression through their unique receptors and pathways. Therefore, an in-depth analysis of the protein structure of the pentraxins family and their underlying mechanisms using advanced electron microscopy technologies, such as cryo-electron microscopy, is important for future research.

The PI3K/AKT/mTOR pathway is commonly associated with the pentraxin family in inducing tumor progression. Nevertheless, each pentraxin family member poses a specific receptor linked with tumor progression. Short pentraxins bind with the Fcγ receptor to activate different pathways. Studies have proposed that the AMPA receptor bridges the neuronal pentraxins and tumor by mediating intracellular free concentration of calcium known to be vital for various downstream pathways. PTX3 modulates tumor cell adhesion and metastasis by interacting with the FGFR system. SAP and PTX4 share highly structural homology with CRP, but limited research has focused on their association with the tumor, similarly with the neuronal pentraxins. Tumor microenvironment and inflammation are two crucial components that influence tumor progression through active communication with each other (138, 139). The pentraxin family can both initiate inflammation and promote tumor progression.

## Author Contributions

QC, ZL, and GX: offered the idea of this review, and acted as the mentors and guarantors of the review. ZW and XW: wrote the review. ZW, XW, and ZD: modified the review. ZW: drew the figures. HZ, MZ, and SF: offered modification advice and checked grammar. All authors contributed to the article and approved the submitted version.

## Conflict of Interest

The authors declare that the research was conducted in the absence of any commercial or financial relationships that could be construed as a potential conflict of interest.
