# Supplementary material for: The Basic Characteristics of the Pentraxin Family and Their Functions in Tumor Progression
Source: Front Immunol. 2020 Aug 18;11:1757. doi: 10.3389/fimmu.2020.01757 (PMC7461825; doi:10.3389/fimmu.2020.01757)
Supplement: Supplementary file 1 [file Data_Sheet_1.ZIP › supplementary figure legend.docx]

**Supplementary figure 1 |** This figure illustrates the outcome of survival analysis predicted by the Gepia database including the neuronal pentraxins (A-M), CRP (N) and PTX4 (O-P). C-reaction protein; APCS: amyloid P component serum; NPTX1: neuronal pentraxin 1; NPTX2: neuronal pentraxin 2; NPTXR: neuronal pentraxin receptor; PTX4: pentraxin 4

**Supplementary figure 2 |** Summarize the outcome of overall survival analysis of PTX3 and tumor. PTX3: pentraxin 3.
