# Supplementary material for: The Basic Characteristics of the Pentraxin Family and Their Functions in Tumor Progression
Source: Front Immunol. 2020 Aug 18;11:1757. doi: 10.3389/fimmu.2020.01757 (PMC7461825; doi:10.3389/fimmu.2020.01757)
Supplement: Supplementary file 1 [file Data_Sheet_1.ZIP › supplementary table.docx]

| Tumor | Statistical value | ACC | BLCA | BRCA | CESC | CHOL | COAD |
| --- | --- | --- | --- | --- | --- | --- | --- |
| CRP | P value | 0.92 | / | 0.72 | / | 0.94 | / |
|  | HR | 0.96 |  | 0.94 |  | 0.97 |  |
| APCS | P value | / | / | / | / | 0.36 | / |
|  | HR |  |  |  |  | 1.6 |  |
| PTX4 | P value | 0.0086 | / | 0.78 | 0.55 | 0.62 | / |
|  | HR | 3.1 |  | 0.95 | 1.2 | 0.77 |  |
| NPTX1 | P value | 0.0028 | 0.012 | 0.98 | 0.59 | 0.45 | 0.16 |
|  | HR | 3.7 | 1.5 | 1 | 0.88 | 0.7 | 1.4 |
| NPTX2 | P value | 0.99 | 0.49 | 0.16 | 0.63 | 0.4 | 0.069 |
|  | HR | 1 | 1.1 | 1.3 | 1.1 | 0.66 | 1.6 |
| NPTXR | P value | 0.57 | 0.23 | 0.33 | 0.21 | 0.79 | 0.028 |
|  | HR | 1.2 | 0.83 | 0.85 | 0.74 | 1.1 | 1.7 |
| PTX3 | P value | 0.11 | 0.14 | 0.038 | 0.011 | 0.23 | 0.29 |
|  | HR | 1.9 | 1.3 | 0.71 | 1.9 | 0.54 | 1.3 |

| Tumor | Statistical value | DLBC | ESCA | GBM | HNSC | KICH | KIRC |
| --- | --- | --- | --- | --- | --- | --- | --- |
| CRP | P value | / | 0.28 | / | 0.061 | / | 0.0004 |
|  | HR |  | 0.77 |  | 1.3 |  | 1.8 |
| APCS | P value | / | / | / | / | / | 0.82 |
|  | HR |  |  |  |  |  | 1 |
| PTX4 | P value | / | 0.66 | 0.18 | 0.0069 | / | 0.17 |
|  | HR |  | 0.89 | 1.3 | 0.67 |  | 0.8 |
| NPTX1 | P value | 0.38 | 0.34 | 0.37 | 0.37 | 0.21 | 0.89 |
|  | HR | 0.52 | 1.3 | 1.2 | 0.88 | 0.4 | 0.98 |
| NPTX2 | P value | 0.18 | 0.41 | 0.007 | 0.31 | 0.37 | 0.089 |
|  | HR | 0.33 | 0.82 | 1.6 | 1.1 | 1.9 | 1.3 |
| NPTXR | P value | 0.75 | 0.98 | 0.75 | 0.57 | 0.62 | 0.14 |
|  | HR | 1.3 | 1 | 1.1 | 1.1 | 0.72 | 1.3 |
| PTX3 | P value | 0.91 | 0.52 | 0.057 | 0.0017 | 0.087 | 0.048 |
|  | HR | 1.1 | 1.2 | 1.4 | 1.5 | 4 | 1.4 |

| Tumor | Statistical value | KIRP | LAML | LGG | LIHC | LUAD | LUSC |
| --- | --- | --- | --- | --- | --- | --- | --- |
| CRP | P value | 0.057 | / | / | 0.81 | 0.18 | 0.85 |
|  | HR | 1.8 |  |  | 1 | 1.2 | 1 |
| APCS | P value | / | / | / | 0.17 | / | / |
|  | HR |  |  |  | 0.78 |  |  |
| PTX4 | P value | / | 0.59 | 0.98 | / | 0.98 | 0.61 |
|  | HR |  | 0.86 | 0.99 |  | 1 | 1.1 |
| NPTX1 | P value | 0.0021 | 0.3 | 0.8 | 0.74 | 0.35 | 0.35 |
|  | HR | 2.8 | 1.3 | 1 | 1.1 | 0.87 | 1.1 |
| NPTX2 | P value | 0.000094 | 0.23 | 0.17 | 0.087 | 0.93 | 0.027 |
|  | HR | 4.4 | 1.4 | 1.3 | 1.4 | 0.99 | 1.4 |
| NPTXR | P value | 0.54 | 0.44 | 0.18 | 0.91 | 0.25 | 0.66 |
|  | HR | 1.2 | 1.2 | 0.78 | 1 | 1.2 | 1.1 |
| PTX3 | P value | 0.01 | 0.34 | 0.00015 | 0.31 | 0.038 | 0.00068 |
|  | HR | 2.3 | 1.3 | 2 | 1.2 | 1.4 | 1.6 |

| Tumor | Statistical value | MESO | OV | PAAD | PCPG | PRAD |
| --- | --- | --- | --- | --- | --- | --- |
| CRP | P value | / | / | 0.2 | / | / |
|  | HR |  |  | 1.3 |  |  |
| APCS | P value | / | / | 0.36 | / | / |
|  | HR |  |  | 1.2 |  |  |
| PTX4 | P value | 0.88 | 0.78 | 0.45 | 0.8 | 0.26 |
|  | HR | 0.96 | 0.96 | 1.2 | 0.79 | 0.46 |
| NPTX1 | P value | 0.063 | 0.4 | 0.32 | 0.37 | 0.13 |
|  | HR | 1.6 | 1.1 | 1.2 | 2.2 | 2.9 |
| NPTX2 | P value | 0.18 | 0.98 | 0.39 | 0.28 | 0.72 |
|  | HR | 1.4 | 1 | 0.84 | 2.6 | 1.3 |
| NPTXR | P value | 0.000081 | 0.69 | 0.017 | 0.95 | 0.17 |
|  | HR | 2.9 | 1.1 | 0.61 | 1.1 | 2.6 |
| PTX3 | P value | 0.026 | 0.61 | 0.79 | 0.37 | 0.75 |
|  | HR | 1.7 | 1.1 | 1.1 | 2.2 | 0.81 |

| Tumor | Statistical value | READ | SARC | SKCM | STAD | TGCT |
| --- | --- | --- | --- | --- | --- | --- |
| CRP | P value | / | 0.079 | 0.46 | / | / |
|  | HR |  | 1.3 | 0.43 |  |  |
| APCS | P value | / | / | / | / | / |
|  | HR |  |  |  |  |  |
| PTX4 | P value | / | 0.82 | 0.7 | 0.83 | 0.5 |
|  | HR |  | 1.1 | 1.1 | 1 | 0.44 |
| NPTX1 | P value | 0.22 | 0.41 | 0.42 | 0.0056 | 0.75 |
|  | HR | 1.9 | 1.2 | 1.1 | 1.6 | 0.72 |
| NPTX2 | P value | 0.31 | 0.067 | 0.42 | 0.098 | 0.47 |
|  | HR | 0.61 | 1.5 | 0.9 | 1.3 | 0.43 |
| NPTXR | P value | 0.21 | 0.42 | 0.24 | 0.29 | 0.49 |
|  | HR | 1.9 | 1.2 | 0.85 | 1.2 | 2.2 |
| PTX3 | P value | 0.85 | 0.66 | 0.13 | 0.023 | 0.44 |
|  | HR | 0.91 | 1.1 | 0.81 | 1.4 | 2.5 |

| Tumor | Statistical value | THCA | THYM | UCEC | UCS | UVM |
| --- | --- | --- | --- | --- | --- | --- |
| CRP | P value | / | / | / | / | 0.57 |
|  | HR |  |  |  |  | 1.4 |
| APCS | P value | / | / | / | / | / |
|  | HR |  |  |  |  |  |
| PTX4 | P value | 0.67 | 0.09 | 0.43 | 0.88 | / |
|  | HR | 0.8 | 4.7 | 0.71 | 0.95 |  |
| NPTX1 | P value | 0.088 | 0.71 | 0.88 | 0.47 | 0.0047 |
|  | HR | 2.7 | 0.77 | 0.95 | 0.78 | 4.8 |
| NPTX2 | P value | 0.21 | 0.33 | 0.065 | 0.98 | 0.0044 |
|  | HR | 2 | 0.49 | 2 | 0.99 | 4.4 |
| NPTXR | P value | 0.46 | 0.27 | 0.043 | 0.27 | 0.5 |
|  | HR | 0.69 | 2.3 | 2.1 | 1.5 | 1.3 |
| PTX3 | P value | 0.013 | 0.55 | 0.02 | 0.1 | 0.073 |
|  | HR | 4.9 | 0.64 | 2.4 | 0.56 | 2.2 |

**Supplementary table |** The statistical data from overall survival analysis of each family members and tumor. CRP: C-reaction protein; APCS: amyloid P component serum;

NPTX1: neuronal pentraxin 1 ; NPTX2: neuronal pentraxin 2; NPTXR: neuronal pentraxin receptor; PTX3: pentraxin 3; PTX4: pentraxin 4; ACC: adrenocortical carcinoma; BLCA: bladder urothelial carcinoma; BRCA: breast invasive carcinoma; CESC: cervical squamous cell carcinoma and endocervical adenocarcinoma; CHOL: cholangiocarcinoma; COAD: colon adenocarcinoma; DLBC: lymphoid neoplasm diffuse large B-cell lymphoma; ESCA: esophageal carcinoma; GBM: glioblastoma multiforme; HNSC: head and neck squamous cell carcinoma; KICH: kidney chromophobe; KIRC: kidney renal clear cell carcinoma; KIRP: kidney renal papillary cell carcinoma; LAML: acute myeloid leukemia; LGG: glioma; LIHC: liver hepatocellular carcinoma; LUAD: lung adenocarcinoma; LUSC: lung squamous cell carcinoma; MESO: mesothelioma; OV: ovarian serous cystadenocarcinoma; PAAD: pancreatic adenocarcinoma; PCPG: pheochromocytoma and paraganglioma; PRAD: prostate adenocarcinoma; READ: rectum adenocarcinoma; SARC: sarcoma; SKCM: skin cutaneous melanoma; STAD: stomach adenocarcinoma; TGCT: testicular germ cell tumors; THCA: thyroid carcinoma; THYM: thymoma; UCEC: uterine corpus endometrial carcinoma; UCS: uterine carcinosarcoma; UVM: uveal melanoma. Some result did not manifest in this table due to sample deficiency according to web portal.
