# Supplementary figures and images for: The Basic Characteristics of the Pentraxin Family and Their Functions in Tumor Progression
Source: Front Immunol. 2020 Aug 18;11:1757. doi: 10.3389/fimmu.2020.01757 (PMC7461825; doi:10.3389/fimmu.2020.01757)

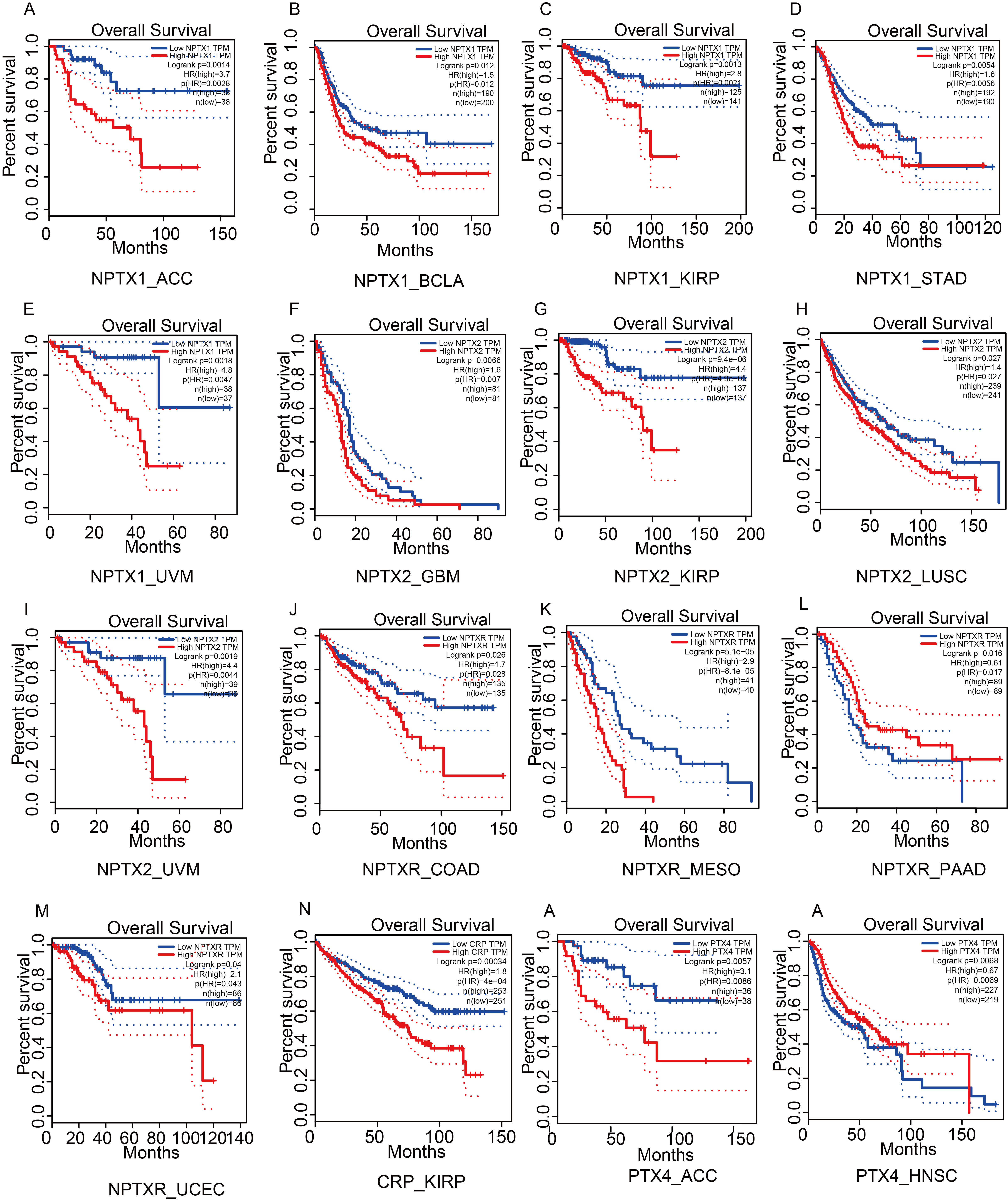

Supplement: Supplementary file 1 [file Data_Sheet_1.ZIP › figure S1.jpg]

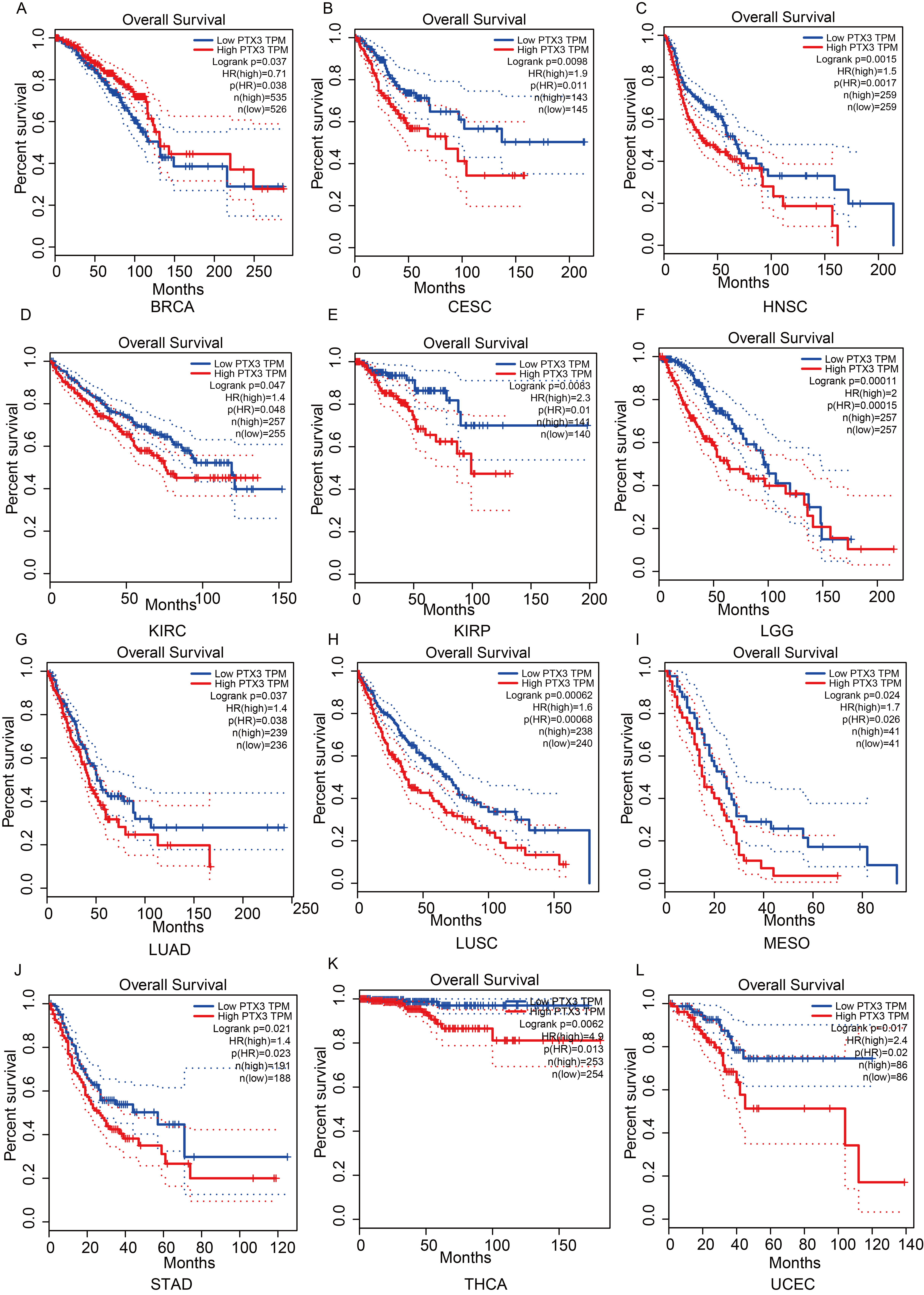

Supplement: Supplementary file 1 [file Data_Sheet_1.ZIP › figure S2.jpg]
